# Estimation of anti-orthopoxvirus immunity in Moscow residents and potential risks of spreading Monkeypox virus

**DOI:** 10.3389/fimmu.2022.1023164

**Published:** 2022-11-16

**Authors:** Vladimir A. Gushchin, Darya A. Ogarkova, Inna V. Dolzhikova, Olga V. Zubkova, Igor V. Grigoriev, Andrei A. Pochtovyi, Anna A. Iliukhina, Tatiana A. Ozharovskaia, Nadezhda A. Kuznetsova, Daria D. Kustova, Artem Y. Shelkov, Denis I. Zrelkin, Alina S. Odintsova, Daria M. Grousova, Vladislav Y. Kan, Sona A. Davtyan, Andrei E. Siniavin, Elizaveta D. Belyaeva, Andrei G. Botikov, Arina A. Bessonova, Lyudmila A. Vasilchenko, Daria V. Vasina, Denis A. Kleymenov, Egor A. Slutskiy, Artem P. Tkachuk, Olga A. Burgasova, Svetlana Y. Loginova, Evgeny V. Rozhdestvensky, Dmitry V. Shcheblyakov, Alexander N. Tsibin, Andrey G. Komarov, Vladimir I. Zlobin, Sergei V. Borisevich, Boris S. Naroditsky, Denis Y. Logunov, Alexander L. Gintsburg

**Affiliations:** ^1^ Department of Science, Federal State Budget Institution “National Research Centre for Epidemiology and Microbiology Named After Honorary Academician N. F. Gamaleya” of the Ministry of Health of the Russian Federation, Moscow, Russia; ^2^ Department of Virology, Biological Faculty, Lomonosov Moscow State University, Moscow, Russia; ^3^ Moscow Healthcare Department, Moscow, Russia; ^4^ Department of Infectious Diseases with the Courses of Epidemiology and Phthisiology, Peoples Friendship University of Russia (RUDN) University, Moscow, Russia; ^5^ Department of Especially Dangerous Viral Infections, 48-Central Research Institute of the Ministry of Defence of the Russian Federation, Moscow, Russia; ^6^ Department of Infectiology and Virology, Federal State Autonomous Educational Institution of Higher Education I.M. Sechenov, First Moscow State Medical University of the Ministry of Health of the Russian Federation (Sechenov University), Moscow, Russia

**Keywords:** monkeypox virus (MPXV), orthopoxvirus, vaccinia virus (VACV), immunity, neutralizing antibody titers (NAT)

## Abstract

WHO has declared the outbreak of monkeypox as a public health emergency of international concern. In less than three months, monkeypox was detected in more than 30 000 people and spread to more than 80 countries around the world. It is believed that the immunity formed to smallpox vaccine can protect from monkeypox infection with high efficiency. The widespread use of Vaccinia virus has not been carried out since the 1980s, which raises the question of the level of residual immunity among the population and the identification of groups requiring priority vaccination. We conducted a cross-sectional serological study of remaining immunity among Moscow residents. To do this, a collection of blood serum samples of age group over 30 years old was formed, an *in-house* ELISA test system was developed, and a virus neutralization protocol was set up. Serum samples were examined for the presence of IgG antibodies against Vaccinia virus (*n*=2908), as well as for the ability to neutralize plaque formation with a Vaccinia virus MNIIVP-10 strain (*n*=299). The results indicate the presence of neutralizing antibody titer of 1/20 or more in 33.3 to 53.2% of people older than 45 years. Among people 30-45 years old who probably have not been vaccinated, the proportion with virus neutralizing antibodies ranged from 3.2 to 6.7%. Despite the higher level of antibodies in age group older than 66 years, the proportion of positive samples in this group was slightly lower than in people aged 46-65 years. The results indicate the priority of vaccination in groups younger than 45, and possibly older than 66 years to ensure the protection of the population in case of spread of monkeypox among Moscow residents. The herd immunity level needed to stop the circulation of the virus should be at least 50.25 – 65.28%.

## Introduction

Poxviruses are widespread in the environment of almost all continents. The genus of orthopoxviruses, in addition to the smallpox virus, includes 12 more representatives. Some of representatives of this genus are zoonoses, however, circulating normally among animals, they can cause diseases in humans. Until recently, most cases of orthopoxvirus infections were sporadic, not leading to epidemic outbreaks and difficult to register (1, 2). At the same time, it is believed that the immunity formed to the smallpox can protect from monkeypox infection at the level of 85% (3).

The greatest concern in the last decade has been caused by the increase in the incidence of monkeypox (4). Thus, the sporadic incidence of monkeypox in Africa over 50 years of observations increased from single cases to thousands per year on average. Considering that the average age of patients also increased from 4 years in the 1970s to 21 in the 2010s, the increase in morbidity is primarily associated with the cessation of mass population immunization. Widespread vaccination was terminated after circulation of the smallpox virus had been stopped. The cessation of mass vaccination for the prevention of smallpox has increased the number of people living in the territory of the natural focus of monkeypox in Africa who do not have an immunity. In fact, people younger 40 years old are not vaccinated, which means they are able to get infected with monkeypox. In addition, it is obvious that over time, the immunity of vaccinated weakens, which can also lead to an increase in the number of cases (5, 6).

Increasing of cases of human-to-human transmission of the virus is the one of the reasons for concern, as well as the appearance of imported cases of monkeypox with the spread of the virus outside the natural foci in Africa (7). Thus, in 2003, 47 people with monkeypox were recorded in the USA (8), and in 2018 there were 4 cases in the UK (9). The greatest concern was caused by the current outbreak of monkeypox, that began in April 2022. In less than three months, monkeypox has been confirmed in more than 30 thousand people, and its geography has spread to more than 80 countries around the world (10). In Russia, one case of monkeypox was also detected (11). As a result, WHO has declared the 2022 outbreak of monkeypox as a public health emergency. Assigning the maximum danger status requires active monitoring of morbidity, preparation of counteraction plans, which should include an assessment of the population protection, expansion of diagnostic capabilities, the use of specific prevention in risk groups, as well as means of specific therapy for patients with monkeypox.

The titer of viral neutralizing antibodies (VNA) in human blood serum are thought to be at least 1/20 to provide protection against smallpox (12–14). In this case, it is considered as preventive virus neutralization titer (PRNT). Studies of residual immunity after receiving the vaccine show that post-vaccination immunity can persist for more than 20 years (13). This makes it possible to expect that at least a part of the population previously vaccinated against smallpox will be able to maintain protection against monkeypox. A study conducted in Brazil in 2016 showed that 53.1% of the population over 35 years old had a PRNT (14). In turn, a 2012 study conducted in China an experimental VNA detection system using a variant of the Vaccinia virus with integrated luciferase showed the presence of a protective titer in 5.5% and 10.3% of age group aged 31-40 years and 41-56 years, respectively (15). Such a different result in the assessment suggests a different response strategy.

The level of residual immunity to smallpox in Moscow is not known. Widespread vaccination of newborns in Moscow, as in most regions of the world, was carried out until 1980. In addition to vaccination of risk groups and newborns, in 1959-1960, after the outbreak of smallpox, more than 10 million doses of the vaccine were used to immunize residents of Moscow and the Moscow region (7). To study residual immunity, we conducted a serological cross-sectional study of blood serum samples in different age group. Serum samples were examined for the presence of IgG antibodies against Vaccinia virus (*n*=2908), as well as for the ability to neutralize plaque formation with Vaccinia virus MNIIVP-10 strain (*n*=299).

## Results

### Samples collection, ELISA system development and the IgG antibodies to Vaccinia virus analysis

We formed a random group of blood serum samples from residents of Moscow from 30 to 80 years old (*n*=2908). Together with the samples, we collected depersonalized data of age in groups 30-35, 36-40, 41-45, 46-50, 51-55, 56-60, 61-65, 66-70, 71-75 and 76-80 years old. Each group included at least 250 patients. Subsequently, serum aliquots were used to determine the level of IgG antibodies to the Vaccinia virus and to assess the VNA against the Vaccinia virus.

Due to the lack of commercially available test systems for the determination of residual IgG antibodies to the Vaccinia virus and standard serum panels, we have developed an in-house laboratory ELISA system for the analysis as described previously (16). It was assumed that the ELISA system uses whole virions of the WR strain, which were obtained during cultivation on Vero E6 cells, followed by purification in a sucrose gradient and confirmation of the conformity of the strain used by genome-wide sequencing (see material and methods). In each assay using the developed system, a negative K- (OD-450 0.065-0.075) and a weakly positive K+ (OD-450 0.200 - 0.240) samples were included in duplicates. To normalize the results, the relative optical density (ODrel) was calculated for each sample, dividing the OD-450 of the sample by the average value of OD-450 K+ in this experiment. The obtained values were conditionally divided into 5 groups: negative (ODrel <0.5), doubtful (0.501 < ODrel < 1.0), weakly positive (1.001 < ODrel. < 2.0), positive (2.001 < ODrel. < 4.0) and highly positive (ODrel > 4.001).

**Figure 1:**
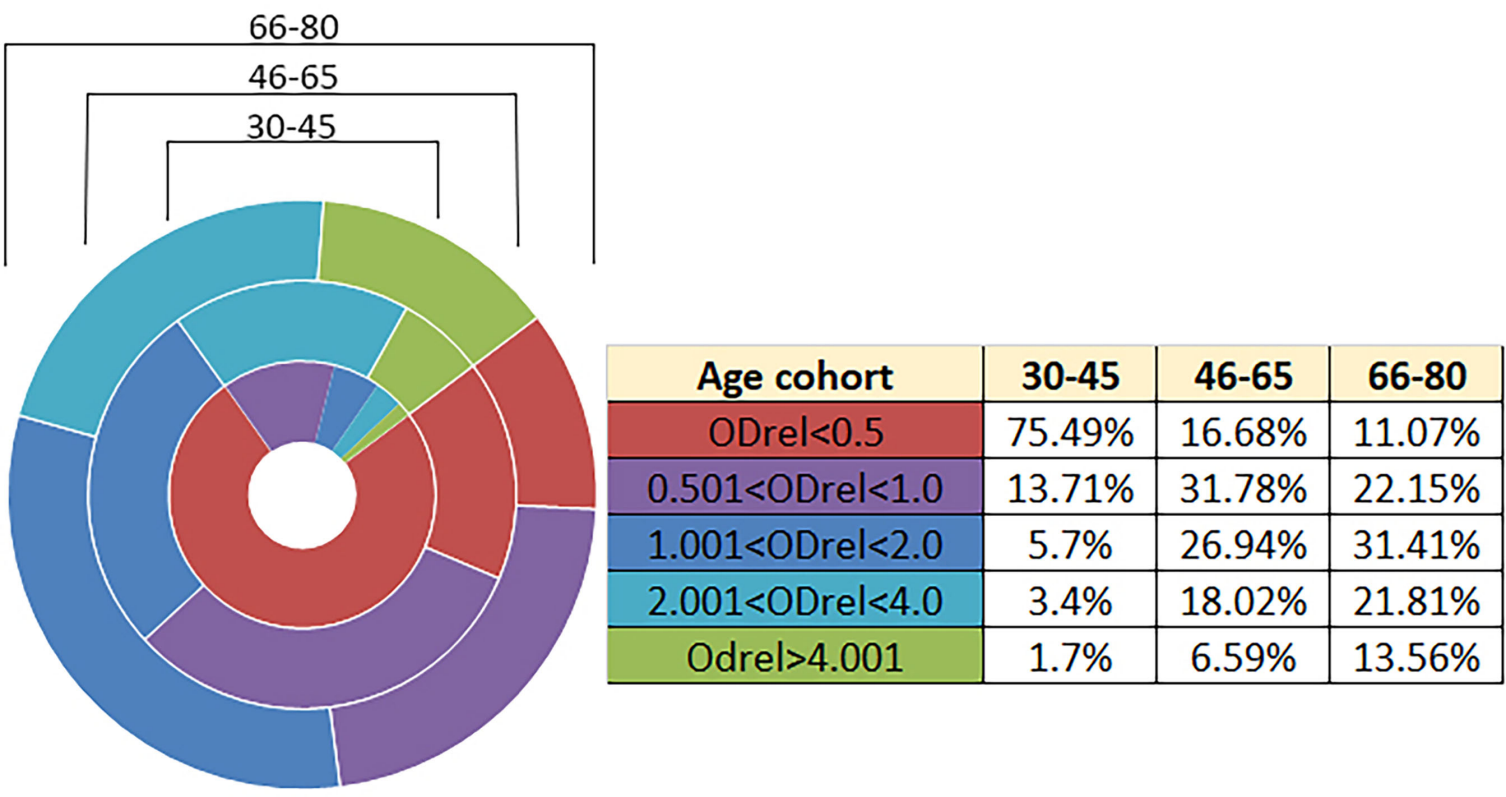
Antibody levels according to age. n = 2908. The relative optical density (ODrel) was calculated for each serum sample, dividing the OD-450 of the sample by the average value of OD-450 K+ (weakly positive control) in this experiment. The obtained values were conditionally divided into 5 groups: negative (ODrel <0.5), doubtful (0.501 < ODrel < 1.0), weakly positive (1.001 < ODrel. < 2.0), positive (2.001 < ODrel. < 4.0) and highly positive (ODrel > 4.001). Percentages have statistically significant differences in any comparisons between ages (max p = 0.031). The maximum percentage of samples obtained from people 30-45 years old showed a negative result in ELISA ((ODrel <0.5), most samples from people 46-65 years old showed doubtful result and the largest number of samples from people 66-80 years old showed a weakly positive result.

According to the results of the analysis using the developed system, we show the dependence of the number of antibodies on the age group (**Table 1**; **Figure 1**). The number of people with a positive response in the ELISA test increases with age. The lowest percentage of seropositive (ODrel > 1.0) is in groups up to 45 years old (about 10.8%). In the middle-aged group of 46-65 years, the percentage of positive samples is 51.55% and reaches maximum values among older people over 66 years old (66.78%) (**Figure 1**).

**Table 1:** Percentages of optical density values for different groups depending on the age of the study participants.

Group	Age groups										Total
	30-35	36-40	41-45	46-50	51-55	56-60	61-65	66-70	71-75	76-80	
Negative (ODrel<0.5)	230 82.4%	207 74.7%	185 69.0%	79 28.4%	40 14.2%	49 16.8%	32 9.2%	36 12.5%	37 11.3%	25 9.2%	920 31.6%
Doubtfull (0.501 < ODrel < 1.0)	26 9.3%	43 15.5%	44 16.4%	94 33.8%	96 34.2%	90 30.9%	101 28.9%	64 22.2%	83 25.5%	49 18.1%	690 23.7%
Weakly positive (1.001 < ODrel < 2.0)	13 4.7%	13 4.7%	21 7.8%	67 24.1%	85 30.2%	67 23.0%	104 29.8%	84 29.2%	102 31.3%	92 33.9%	648 22.3%
Positive(2.001 < ODrel < 4.0)	9 3.2%	8 2.9%	11 4.1%	32 11.5%	46 16.4%	66 22.7%	72 20.6%	65 22.6%	72 22.1%	56 20.7%	437 15.0%
Highly positive (ODrel > 4.001)	1 0.4%	6 2.2%	7 2.6%	6 2.2%	14 5.0%	19 6.5%	40 11.5%	39 13.5%	32 9.8%	49 18.1%	213 7.3%
Total	279	277	268	278	281	291	349	288	326	271	2908

### Analysis of virus neutralizing antibodies in blood serum samples using Vaccinia virus

**Figure 2:**
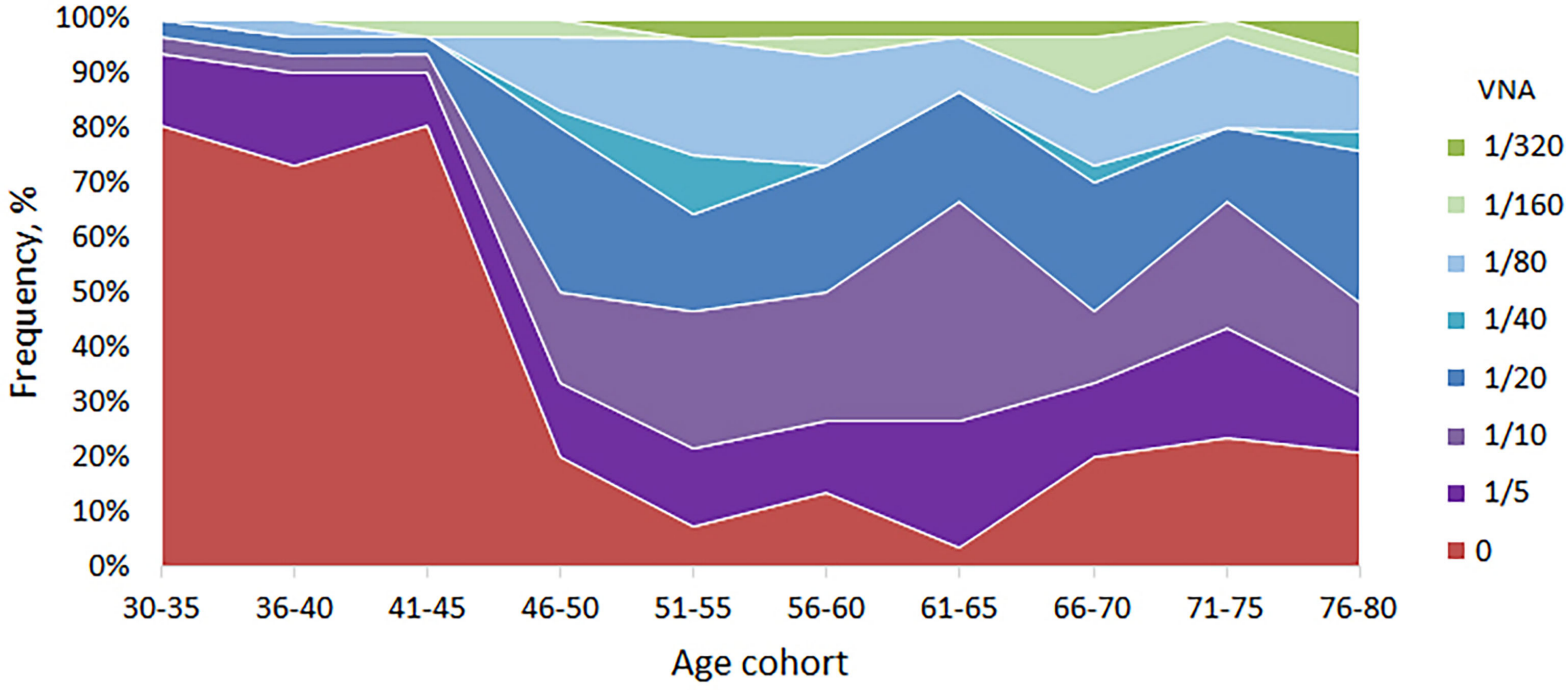
VNA in different age cohorts. The frequency of samples with VNA = 0 drops sharply in age cohorts older than 45 years. For people younger than 45 years old, the proportion of people with VNA ≥ 1/20 titer ranged from 3.2 to 6.7%, and for people older than 45 years old – from 33.3 to 53.2%.

Having established the basic patterns of the distribution of antibodies depending on the age of Moscow residents, we studied the VNA in relation to the laboratory strain of smallpox vaccine. To do this, an adapted method was used to assess the reduction of plaque formation in the presence of various serum dilutions. We used dilutions 1/5, 1/10, 1/20, 1/40, 1/80, 1/160 and 1/320. Titers 1/20 and higher were accepted by us as PRNT. The analysis of viral neutralizing activity was carried out for randomly selected 299 serum samples. For 104 (34.8%) samples, viral neutralizing activity was not found, for 44 (14.7%) – 1/5, for 50 (16.7%) - 1/10 and for 101 (33.8%) – 1/20 or more. The distribution of VNA in relation to age is shown in **Table 2** and **Figure 2**.

**Table 2:** Virus neutralizing activity for Vaccinia virus in dependence of the age.

Age group	VNA (max dilutions with neutralizing activity, 1/х)								
	0	5	10	20	40	80	160	320	*n*
**30-35**	25 (80.6%)	4 12.9%)	1 (3.2%)	1 (3.2%)	0 (0%)	0 (0%)	0 (0%)	0 (0%)	31
**36-40**	22 (73.3%)	5 (16.7%)	1 (3.3%)	1 (3.3%)	0 (0%)	1 (3.3%)	0 (0%)	0 (0%)	30
**41-45**	25 (80.6%)	3 (9.7%)	1 (3.2%)	1 (3.2%)	0 (0%)	0 (0%)	1 (3.2%)	0 (0%)	31
**46-50**	6 (20.0%)	4 (13.3%)	5 (16.7%)	9 (30.0%)	1 (3.3%)	4 (13.3%)	1 (3.3%)	0 (0%)	30
**51-55**	2 (7.1%)	4 (14.3%)	7 (25.0%)	5 (17.9%)	3 (10.7%)	6 (21.4%)	0 (0%)	1 (3.6%)	28
**56-60**	4 (13.3%)	4 (13.3%)	7 (23.3%)	7 (23.3%)	0 (0%)	6 (20.0%)	1 (3.3%)	1 (3.3%)	30
**61-65**	1 (3.3%)	7 (23.3%)	12 (40.0%)	6 (20.0%)	0 (0%)	3 (10.0%)	0 (0%)	1 (3.3%)	30
**66-70**	6 (20.0%)	4 (13.3%)	4 (13.3%)	7 (23.3%)	1 (3.3%)	4 (13.3%)	3 (10.0%)	1 (3.3%)	30
**71-75**	7 (23.3%)	6 (20.0%)	7 (23.3%)	4 (13.3%)	0 (0%)	5 (16.7%)	1 (3.3%)	0 (0%)	30
**76-80**	6 (20.7%)	3 (10.3%)	5 (17.2%)	8 (27.6%)	1 (3.4%)	3 (10.3%)	1 (3.4%)	2 (6.9%)	29
**Total**	104 (34.8%)	44 (14.7%)	50 (16.7%)	49 16.4%)	6 (2.0%)	32 10.7%)	8 (2.7%)	6 (2.0%)	299

VNA varied depending on the age cohort. So, for people younger than 45 years old, the proportion of people with VNA ≥ 1/20 titer ranged from 3.2 to 6.7%, and for people older than 45 years old – from 33.3 to 53.2%. The differences were significant and make it possible to distinguish well between people under and over 45 years old (**Table 3**). Also, high VNA (more than 1/160 dilutions) was shown for people older than 45 years (**Table 2**). For people younger than 45 years virus neutralizing activity in dilutions 1/40, 1/80, 1/160 was found in sporadic cases.

**Table 3:** The significance of differences in the proportion of people with VNA ≥ 1/20 depending on the age cohort.

Age cohort	30-35	36-40	41-45	46-50	51-55	56-60	61-65	66-70	71-75	76-80
**30-35**	–	0.752	0.755	<0.001*	<0.001*	<0.001*	0.006*	<0.001*	0.006*	<0.001*
**36-40**		–	1.000	0.001*	<0.001*	0.001*	0.021*	<0.001*	0.021*	0.001*
**41-45**			–	0.001*	<0.001*	0.001*	0.020*	<0.001*	0.020*	<0.001*
**46-50**				–	0.968	1.000	0.276	0.968	0.276	0.989
**51-55**					–	0.968	0.216	1.000	0.216	0.989
**56-60**						–	0.276	0.968	0.276	0.989
**61-65**							–	0.216	1.000	0.255
**66-70**								–	0.216	1.000
**71-75**									–	0.255
**76-80**										–

The chi-square criterion with Benjamin Hochberg adjusting. An asterisk indicates statistically significant differences (*p*<0.05).

**Figure 3:**
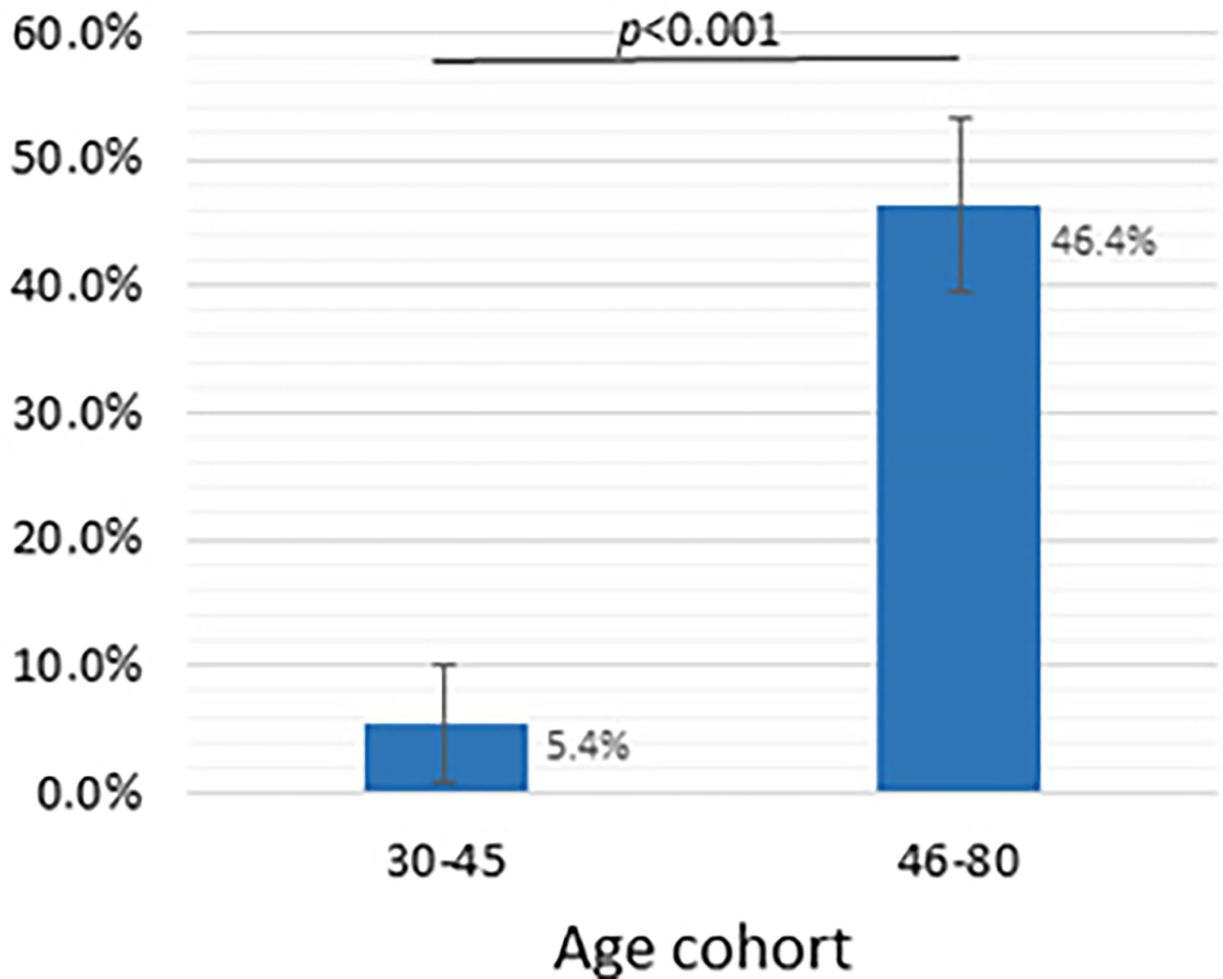
Dependence of the presence of viral neutralizing activity of blood serum sample on the age group, dilution - 1/20 and more. Y-axis shows the frequency of samples with VNA ≥ 1/20 (%), whiskers show a 95% confidence interval calculated by the Wald method. Two groups compared using chi-square test.

We combined the age cohorts into two groups: (i) the 30-45-year–old cohort (VNA ≥ 1/20 dilutions was found in 5 out of 92 people, which was 5.4% (95% CI: 0.8% - 10.0%)), and (ii) 46-80-year-old cohort (VNA ≥ 1/20 dilutions was found in 96 out of 207, which was 46.4% (95% CI: 39.6% - 53.2%)). The values are shown in **Figure 3**.

### Comparison of methods for assessing residual immunity of ELISA and VNA

Having received two data arrays with the values of the ODrel ELISA and the VNA titer, we analyzed the correspondence of the values obtained by two independent methods. So, in any of the two age cohorts (30-45 and 46-80 years) the level of antibodies was significantly higher in the group that showed high viral neutralizing activity (VNA ≥ 1/20, **Table 4**). The level of antibodies to Vaccinia virus in the group 46-80 years old was significantly higher at any values of VNA and increased with an increase in the VNA titer (*p*<0.05 for all comparisons). In turn, in the 30-45-year-old group, the values of ELISA differed significantly only for groups with VNA ≥ 1/20 (*p*=0.003 and *p*=0.011 in comparison with VNA=0 and 1/5 ≤ VNA ≤ 1/10 respectively). This may indicate that decreasing both in the titer of IgG and VNA in previously vaccinated patients in the 46-80-year-old group occurs gradually.

**Table 4:** Median trends in the number of antibodies to orthopoxviruses (ODrel, Me[IQR]) depending on age and VNA.

Age cohort	VNA (max dilutions with neutralizing activity)			
	1.	2.	3.	*p* (Kraskell Wallis criterion)
	0	1/5 – 1/10	≥ 1/20
30-45	n = 72 0.31 [0.29 – 0.40]	n = 15 0.32 [0.28 – 0.46]	n = 5 0.92 [0.59 – 2.98]	0.004* p (1vs2) = 1.000 p (1vs3) = 0.003* p (2vs3) = 0.011*
46-80	n = 32 0.49 [0.36 – 0.85]	n = 79 0.94 [0.54 – 1.54]	n = 95 1.92 [1.04 – 2.88]	<0.001* p (1vs2 ) = 0.011* p (1vs3)<0.001* p (2vs3)<0.001*
*p* (Mann Whitney criterion)	<0.001*	<0.001*	0.411	

*differences are statistically significant (p<0.05).

**Figure 4:**
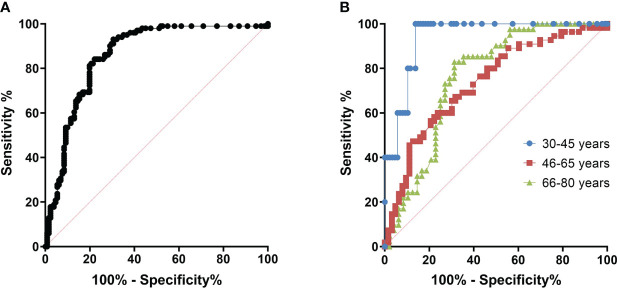
ROC curves for determining the cut off at which it is possible to predict viral neutralizing activity. **(A)** – All age cohorts, *n* = 299, AUC = 0.833 (95% CI: 0.787 - 0,880), *p*<0.001; **(B)** – With division by age/Blue – 30-45 years old (*n*=92), AUC = 0.940 (95% CI: 0.879 – 1.000), *p*=0.001; Red – 46-65 years old (*n*=118), AUC=0.734 (95% CI: 0.643 – 0.824), *p*<0.001; Green – 66-80 years old (*n*=89), AUC=0.759 (95% CI: 0.658 – 0.860), *p*<0.001.

We have constructed ROC curves to determine the ODrel value of antibodies to the Vaccinia virus, at which we can assume the presence of a protective titer of VNA ≥ 1/20. In **Figure 4** we showed the ROC-curve for all participants and for age groups: 30-45, 46-65 and 66-80 years of age. For all cases ROC-curves were significant (*p*<0.05, see **Figure 4**).

The area under the curve was 0.940 (95% CI: 0.879 – 1,000) for the 30-45–year-old cohort, 0.734 (95% CI: 0.643 - 0.824) for the 46-65-year-old cohort and 0.759 (95% CI: 0.658 – 0.860) for 66-80-year-old cohort. The cut-off point was chosen with ROC-curve for all cohorts (**Figure 4A**) as more accurate. Assuming that in the case of an ODrel value ≥ 0.995, the serum has a viral neutralizing activity (VNA ≥ 1/20), the sensitivity was 75.2%, and the specificity was 75.3%. Positive prognostic value was 60.8% and negative prognostic value was 85.6%.

Extrapolation of the results for the remaining patients for whom the VNA analysis was not performed, the probability of viral neutralization was predicted based on information about the presence of antibodies in ELISA (**Table 5**). The analysis shows that it is possible to distinguish two key age groups - 30-45 years, for which the percentage of presumably having VNA does not exceed 15.6%, and the group older than 46, for which the proportion of persons with protective titer changes with age from 39.1 to 73.1%.

**Table 5:** Predicted VNA by age according to ODrel, n = 2610.

Year cohort	Odrel<0.995 (predicting VNA<1/20)	ODrel ≥ 0.995 (predicting VNA ≥ 1/20)
30-35	228 (91.9%)	20 (8.1%)
35 – 40	222 (89.9%)	25 (10.1%)
41 – 45	200 (84.4%)	37 (15.6%)
46 – 50	151 (60.9%)	97 (39.1%)
51 – 55	123 (48.6%)	130 (51.4%)
56 – 60	124 (47.5%)	137 (52.5%)
61 – 65	122 (38.2%)	197 (61.8%)
66 – 70	93 (36.5%)	188 (63.5%)
71 – 75	108 (36.5%)	188 (63.5%)
76 – 80	65 (26.9%)	177 (73.1%)

Considering the increasing in the number of antibodies with age in the presumably vaccinated and those who probably received additional immunization in Moscow in 1959-1960, we tested the hypothesis of an increase in VNA and ODrel to the Vaccinia virus in the cohort of 66-80 years old relative to 46-65 years old (**Table 6**).

**Table 6:** The level of antibodies and VNA in the age cohorts 46-65 and 66-80 years.

	Age cohort		*p* (criterion)
	46 – 65	66 – 80
**ODrel** **Me[IQR], n = 2074**	*n* = 1199 1.03 [0.61 – 1.96]	*n* = 885 1.43 [0.80 – 2.62]	<0.001* (Mann Whitney criterion)
**VNA, 1/x, n = 207**	*n* = 118	*n = 89*	0.333 (exact Fisher-s test)
**0, n (%)**	13 (11.0%)	19 (21.3%)	
**5, n (%)**	19 (16.1%)	13 (14.6%)	
**10, n (%)**	31 (26.3%)	16 (18.0%)	
**20, n (%)**	27 (22.9%)	19 (21.3%)	
**40, n (%)**	4 (3.4%)	2 (2.2%)	
**80, n (%)**	19 (16.1%)	12 (13.5%)	
**160, n (%)**	2 (1.7%)	5 (5.6%)	
**320, n (%)**	3 (2.5%)	3 (3.4%)	

*differences are statistically significant (p<0.05).

**Figure 5:**
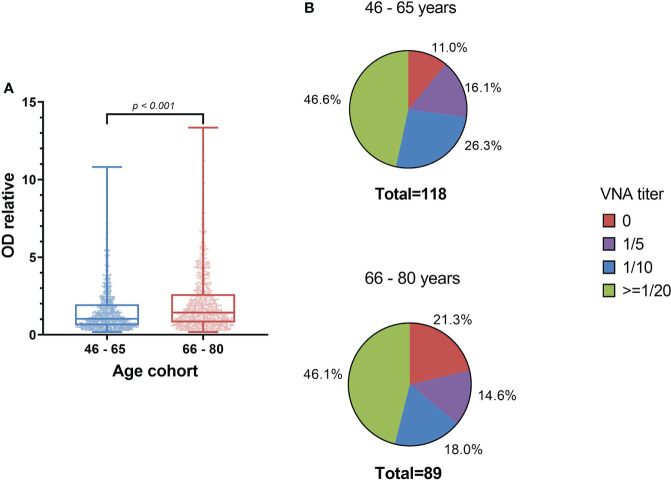
The level of antibodies to smallpox vaccine depending on the age cohort **(A)** and the titer of the VNA **(B)**. **(A)** shows significant differences in IgGs level between samples from 46-65 years old people and 66-80 years old people (Mann Whitney criterion). Whiskers shows maximum and minimum. **(B)** shows VNA titer ratio in 46-65 and 66-80 years old. *p*=0.333 (exact Fisher’s test).

The level of antibodies increases with age, for example, in the age cohort of 66-80 years, ODrel significantly higher than in cohort 46-65 years old (*p* < 0.001). The median relative optical density was 1.43 [0.80 – 2.62] in the older age cohort and 1.03 [0.61 – 1.96] in the 46-65-year-old group. At the same time, no significant increasing in VNA was detected (*p* = 0.333). The proportion of persons with VNA ≥ 20 is 46.6% (*n*=55) in the age cohort of 46-65 years old and 46.1% (*n*=41) in the age cohort of 66-80 years old (**Figure 5**). Thus, despite the greater level of antibodies in 66-80 years old people, their VNA not increase.

### Calculation of the necessary herd immunity level to protect against monkeypox

The Herd immunity is the phenomenon of stopping the spread of an infection when a certain proportion of immune (unsusceptible to infection) individuals is reached and the reproductive number (R0 - the average number of people infected from an infected person) drops below one. This leads to the fact that the number of cases no longer increases over time and begins to decline. The higher the R0, the greater the proportion of immune individuals required to achieve herd immunity. To calculate minimal level of herd immunity among the population, it is necessary to know the reproduction index of the monkeypox virus. Since an insufficient number of cases have been registered in the Russian Federation, CDC statistics on cases identified in the United States (17) or the worldwide data (10) can be used for counting. Seven-days moving average was used to offset the influence of random factors associated with daily case accounting. To calculate R0, we took the period when the moving average overcame 10 cases/day. For global statistics, the observation period was 20.05 – 27.07, for the USA - 22.06 – 25.07. R0 was calculated using the EpiEstimate package in the R programming environment, an incubation period of 9.8 days with a standard deviation of 4 days was used for calculation (18). R0 was calculated with a shift of 7 days. The R0 obtained in this way are shown in **Figure S1**.

The maximum value of R0 was 8.97 for the world and 7.99 for the USA. The median was 1.61 and 2.63 for the world and the USA, respectively, and the average was 2.01 (95% CI: 1.67 – 2.35) and 2.88 (95% CI: 2.24 – 3.51). Averages R0 was used to calculate the immune layer required to stop virus circulation among people. This Herd immunity level was calculated by formula H = 1-(1/R0) and should be 50.25% - 65.28%. Considering the peculiarities of the spread of the monkeypox virus, it is probably possible to expect high R0 at the beginning of the outbreak associated with any social activities accompanied by an increased risk of spread, followed by a sharp decrease in the reproduction index.

## Discussion

The termination of the mass vaccination program against smallpox greatly increased the spread of the monkeypox virus into the human population (4). The growth of sporadic morbidity on the territory of natural foci in Africa invariably led to the appearance of imported cases in developed countries. The epidemic outbreak of 2022 outside the natural focus, considering the geography with a spread on the territory of 80 countries, turned into a pandemic. More than 30 thousand cases of the disease have been recorded worldwide. WHO has declared the outbreak of monkeypox as a public health emergency. A significant risk of widespread virus circulation in the human population is a possible genetic adaptation to more efficient aerogenic transmission of the virus from person to person (19). Genetic data indicate significant changes in the genome structure of the spreading variant of the monkeypox virus compared with previously known cases of virus introduction into the human population in Africa (20). The potential circulation of monkeypox in the population of HIV-positive individuals with immunodeficiency creates additional risks of accelerating the appearance of variants of the virus capable of increased transmission from person to person or causing a more severe course of the disease (21–23).

Some of European states have deployed vaccination using the replicatively incompetent live vaccine JYNNEOS of the Danish company Bavarian Nordic (Smallpox and Monkeypox Vaccine, Live, Non-Replicating) (24). Considering the insufficient volume of the vaccine, it is planned to be used in risk groups. The identification of groups for priority vaccination can be carried out not only considering risk factors, but also considering the residual level of immunity formed by the mass vaccination program. Residual immunity at the VNA level above 1/20 is considered protective against smallpox (13). Since the smallpox immunization program in Africa for a long-time controlled cases of monkeypox with an efficiency of 85% (3) this protective level can be used as a surrogate marker of protection against monkeypox, until a better marker is found.

In the study, two markers of residual immunity to Vaccinia virus were used in Moscow residents. We assessed the residual level of IgG antibodies to Vaccinia virus, as well as the VNA titer in various age groups, including 30-35, 36-40, 41-45, 46-50, 51-55, 56-60, 61-65, 66-70, 71-75 and 76-80 years old. In general, the number of people with IgG antibodies to Vaccinia virus increased with age. Two age transitions of 45 and 66 years old attract attention. The first transition to 45 years and older is obvious. Since people under 45 probably have not received the vaccine, among them about 3.2 – 6.7% have VNA ≥ 1/20, and the protective level of antibodies (ODrel > 1.001) have 10.8% pople. The presence of protective immunity in this group has already been described earlier. Thus, a 2016 Brazilian study showed the presence of protective immunity in 6.2% of persons under 35 years of age (14). The presence of such a group may indicate the possibility of vaccination of these citizens according to indications (military, doctors), or contact with orthopoxviruses on the territory of natural foci (travelers, rural residents).

In the group of people over 45 years of age we observed from 33.3 to 53.2% people with protective immunity to the Vaccinia virus from all people in this age group. There is a significant increase in the level of antibodies in the group of 66-80 years old compared with 46-65 years old group. This increase may be explained by additional immunization, which was carried out in Moscow during the outbreak of smallpox in 1959-1960 (25). During this period, up to 10 million doses were used to vaccinate Moscovites and residents of the Moscow region. Also, in the older group, there is a greater chance of contact with orthopoxviruses, which can increase the level of antibodies. In addition, it is likely that the level of antibodies to the Vaccinia virus could be affected by potentially more frequent contact with the orthopoxviruses that periodically circulated in the past, as well as contact with Vaccinia virus because of vaccination of their own children and grandchildren. Thus, it is known that the virus is released into the environment because of the appearance of pustules in vaccinated (25). Interestingly, an increase in IgG levels in older people to smallpox vaccine does not lead to a statistically significant increase in the VNA titer in our study. One of the factors of the observed phenomenon may be the aging of the immune system of older patients (26, 27)

The results indicate almost complete vulnerability to the threat of the spread of monkeypox of persons under the age of 45. Also, a decrease in the protective titer of VNA in persons over 66 years of age causes concern. This indicates the relevance of the priority use of vaccination in these groups. The availability of safe vaccines remains a problem. In the Russian Federation, two drugs have been registered for medical use that are most likely to protect against monkeypox. The one is the medication for primary immunization - live smallpox vaccine (Smallpox vaccine) (Smallpox vaccine live (smallpox vaccine)) (produced by AO Microgen) (28). The second one is the drug for adult revaccination TEOVac (live smallpox embryonic vaccine) (produced by 48-Central Research Institute of the Ministry of Defence Russia) (29).

Both vaccines cannot be widely recommended as primary immunization agents for the prevention of monkey pox (25). Their use is limited in chronic diseases, pregnant women, and people with immunodeficiency. As a safer alternative, smallpox inactivated vaccine can be used (30). An inactivated vaccine with significantly lower reactogenicity does not cause a prolonged immune response. Its use is advisable at the first stage during two-stage smallpox vaccination followed by vaccination with live smallpox vaccine (25). In this regard, the issue of developing and creating a reserve of third- and fourth-generation vaccines with high efficacy against orthopoxviruses and monkeypox virus as well as low reactogenicity for the possibility of their widespread use is acute.

In the study we showed the presence of a significant stratum of people with a protective level of VNA after more than 40 years after vaccination. This is a significantly longer period than was previously known (13). In cohorts of people over 45 years old, from 33.3% to 53.6% have a protective level of antibodies. The proportion with protective titers was significantly higher than described for China (15) and comparable to the proportion obtained for residents of Brazil (14), USA (31, 32), Japan (33) and Italy (2).

The advantage of our study is many analyzed samples, detailing cohorts by age and using not only the ELISA method, but also in combination with the test of viral neutralizing activity on a laboratory strain of Vaccinia virus. The established level of protection is insufficient to achieve collective immunity. Our calculations based on open data on the incidence of monkeypox in 2022 show that to stop the circulation of the virus in the population, at least 50.25 – 65.28% must be immune to monkeypox. According to Gani and Leach (34) R0 of smallpox virus can vary from 3.5 to 6, so the minimal herd immunity level on the start of epidemic process may reach 71.4 – 83.3%.

Our study has several limitations because we did not evaluate the protective level against monkeypox virus. In the Russian Federation, the monkeypox virus is classified as pathogens of the first group of pathogenicity (BSL 4), which significantly limits research. The presence of high VNA titers for the Vaccinia virus may not fully reflect the protection against monkeypox. Literature data indicate that the cross-immunity among orthopox-virus (including Vaccinia virus, Smallpox, Monkeypox etc.) is so strong that it allows us to count on successful extrapolation of the data obtained for protection against monkeypox virus (35, 36).

## Conclusion

In the age group under 45 years of age, the proportion of people with a protective level of antibodies does not exceed 6.7%. Among people over 45, it ranges from 33.3 to 53.2%. An increase in the level of IgG antibodies to the Vaccinia virus among people older than 45 years of age does not lead to an increase in the protective level in VNA. On the contrary, there may be a tendency to decrease the protective level. Priority for vaccination by age are groups of people under 45 years old, as well as, possibly, over 66 years old. To ensure the protection of the population in the event of widespread monkeypox in Moscow, it is necessary to introduce an effective and low-reactogenic vaccine of a new generation. To stop the circulation of the virus in the population, it is necessary to achieve collective immunity at a level of at least 50.25 – 65.28%.

## Materials and methods

### Collection of serum samples

The purpose of this study was to assess the residual level and intensity of collective immunity against orthopoxviruses in people living in the city of Moscow. During the study, sample blood sera collection over 30 years old was carried out from June 2 to 16 by the Diagnostic Center for Laboratory Research of the Department of Health of the City of Moscow, in total, blood serum samples were collected from 3216 persons. 111 of them had no age information and were excluded from further analysis. Blood serum of 2908 people included in the study was analyzed for the presence of IgG antibodies to Vaccinia virus. The virus neutralizing activity study was conducted for 299 of them. The algorithm for preparing the database for analysis is shown in **Figure S2**. Blood serum samples were the remains of biological materials sent for other purposes of the study. Standard written informed consent was obtained from all subjects allowing the use of leftovers for research purposes. No personal information had been used.

### Cell lines

Vero E6 cells (African marmoset kidney epithelial cells) for NtAb analysis were cultured in DMEM (Cytiva) with 2% heat-inactivated fetal bovine serum (HI-FBS) (Capricorn). The cells were infected at moi 0.1, after 72 hours the cells and the medium were collected, refrozen three times, the debris was precipitated at 9000g for 10 minutes, and the supernatant was aliquoted and stored at -80°C.

### Vaccina virus purification

The Vaccinia virus strain Western Reserve WR strain was grown on Vero E6 cells in T175 cm^2^ tissue culture flask. A flask with a confluent monolayer of cells was infected with the smallpox vaccine virus at a dose of 1-5 PFU/cell. At the onset of 90-100% cytopathic effect, infected cells were collected and destroyed by triple freezing (-70° C) and thawing (+37° C) to release the virus from the cells. The lysates were centrifuged at 2000 rpm at 4°C for 10 minutes, the sediment was removed. Purification and concentration of the smallpox vaccine virus was carried out by ultracentrifugation through a 30% tris-sucrose buffer (10 mM Tris-HCl, 1mM EDTA, 30% sucrose) (Sigma-Aldrich, USA) on an Optima XPN ultracentrifuge (Beckman Coulter Inc., USA) for 70 minutes at 27000 rpm and 14°C in the SW28 rotor. The supernatant was removed, and the precipitate was dissolved in a phosphate buffer. The purified preparation of the smallpox vaccine virus was titrated by plaque formation assay. To do this, 3 x 10^6^ Vero E6 cells were seeded on a 60 mm x 15 mm culture dish. A day later, the cells were infected with tenfold dilutions of the viral stock diluted in DMEMx1 medium without the addition of serum and incubated in a CO2 incubator under the above conditions for 72-96 h. After that, the cells were stained with a neutral red solution (Sigma-Aldich, USA; 3.3 mg/ml). The number of plaques was calculated using a CKX41SF microscope (Olympus, Japan).

Viral DNA was isolated from a purified sample of the smallpox vaccine virus by the Wizard Genomic DNA Purification Kit (Promega, USA). 500 µl of “Nuclei Lysis Solution” and 5 µl of proteinase K (Syntol, Russia) with a concentration of 10 mg/ml were added to 200 µl of the virus (Syntol, Russia). The solution was stirred on a vortex for 5 seconds and placed in a thermostat for 20 minutes at a temperature of 65° C. Then 200 ml of “Protein Precipitation Solution” was added to it. The solution was stirred on a vortex for 5 seconds and placed on ice for 5 minutes, centrifuged at 13,000 rpm for 10 minutes at 4° C. 600 µl of isopropanol was added to the supernatant (Sigma-Aldich, USA), centrifuged at 13,000 rpm for 30 minutes at 4°C. The precipitate was washed with 70% ethanol (Reachim, Russia), dried in air and dissolved in water.

### Sequencing of Vaccinia virus

The genome of vaccinia virus strain was sequenced using Oxford Nanopore Technologies. Sequencing libraries were prepared using SQK-LSK109 (Oxford Nanopore Technologies, Oxford, UK) and native barcoding expansion kit EXP-NBD114 (Oxford Nanopore Technologies, Oxford, UK). The final library was loaded onto an R9.4.1 flow cell and was run for 24 hours. The run was performed on MinION (Oxford Nanopore Technologies, Oxford, UK) using MinKNOW v21.11.8.

### Bioinformatics analyses

Raw nanopore data were basecalling using Guppy v5.1.13 with high accuracy parameter. Fastq file was demultiplexed using Guppy v5.1.13. The demultiplexed long reads were filtered to remove reads under 1000 bases in length using NanoFilt v2.8.0. The adapter trimmed, filtered long reads were mapped to Vaccinia virus (GenBank Accession AY243312.1) using minimap2 v2.17-r941 to remove host and non-Vaccinia virus reads. Data received were *de novo* assembled using Flye v2.9-b1768. Long reads were mapped to the assembly using minimap2 v2.17-r941, and the resulting alignment file was used to polished with the same long read set using Medaka v1.0.3.

The final sequence of this vaccinia virus strain was compared to all sequences available in the NCBI database by using BLASTn. Comparative analysis showed 100% coverage and 99.8% identity with Vaccinia virus strain WR (Accession GenBank AY243312.1).

### Obtaining an ELISA system and assessing the level of IgG antibodies to the vaccinia virus

To obtain the antigen, the cell precipitate was lysed and purified by ultracentrifugation in a stepwise sucrose gradient. For ELISA, 96-well Costar high binding (Corning) plates were sorbed with a virus preparation at a dilution of 1:250 (107pfu/ml) in carbonate buffer pH 9.6 overnight at +4C°. Then the non-bound antigen was removed, and free binding sites were blocked with S002X buffer (Xema, Russia) with 1% casein for an hour at room temperature, after which the plates were stored at +4°C before use. Serum samples at a dilution of 1:400 in buffer S011 (Xema, Russia) were introduced into the wells and incubated for 1 hour at +37°C and stirring 600 rpm. After that, the plates were washed three times with PBS with 0.1% Tween-20 and incubated with anti-human IgG-HRP conjugate (Abcam A18823) in a dilution of 1:50,000 for an hour under the conditions described above. After incubation with the conjugate, the plates were washed 5 times and incubated with a substrate buffer, containing tetramethylbenzidine (R055, Xema) for 15 minutes at room temperature. The reaction was stopped with a stop solution and the optical density was measured at a wavelength of 450 nm.

In each experiment using the developed system, a negative K- (OD-450 0.065-0.075) and a weakly positive K+ (OD-450 0.200 - 0.240) samples were included in duplicates. To normalize the results, the relative optical density (ODrel) was calculated for each sample by dividing the OD-450 of the sample by the average value of OD-450 K+ in this plate/experiment. The obtained values were conditionally divided into 5 groups: negative (ODrel <0.5), doubtful (0.501 < ODrel < 1.0), weakly positive (1.001 < ODrel. < 2.0), positive (2.001 < ODrel. < 4.0) and highly positive (ODrel > 4.001).

### Determination of neutralizing antibody titers

The NtAb level in the sera samples was determined by the plaque reduction neutralization test (PRNT). The sera were inactivated at 56°C for 30 minutes, then two-fold dilutions were prepared in DMEM with 2% HI-FBS, 100 µl of serum dilutions were mixed with 100 µl of vaccinia virus (strain MNIIVP-10) suspension (1000 PFU/ml), incubated at 37°C for 60 minutes and added to monolayer of Vero E6 cells. The serum-virus mix was removed after 4 hours, and cells were overlaid with 0.7% carboxymethylcellulose (CMC; Sigma, USA). After 72 hours, the cells were fixed with 44% Paraformaldehyde (Sigma, USA) and stained with 1% crystal violet (1xPBS, 20% EtOH) and the number of plaques was counted visually. The titer of neutralizing antibodies was considered the maximum dilution of serum, in which a decrease in the number of plaques by more than 50% relative to the control was detected.

### Statistical analysis

Statistical analysis was carried out using the RStudio environment for working in the R programming environment, SPSS Statistics ver. 26 (IBM, USA) and GraphPad Prism (GraphPad Software Inc, California).

Before the analysis of quantitative features, the normality of the distribution was checked using the Shapiro Wilk criterion. In most age groups, the distribution was significantly different from normal (p<0.05), so nonparametric metrics were used to describe the groups – median, first and third quartiles.

We compared groups with the Kruskal Wallis Criterion, followed by pairwise comparisons using the Mann Whitney criterion with Bonferroni adjustment. The analysis of qualitative features was carried out with the construction of conjugacy tables, followed by an assessment of the significance of differences using the chi-square criterion with Bonferroni adjustment if needed. 95% confidence intervals calculated using Wald method.

The cut-off points for determining the level of antibodies at which the presence of viral neutralizing activity was assumed was considered using the construction of ROC curves (SPSS Statistics) as the point at which sensitivity is approximately equal to specificity.

Seven-days moving average was used to offset the influence of random factors associated with daily case accounting. R0 was calculated using the EpiEstimate package in the R programming environment, an incubation period of 9.8 days with a standard deviation of 4 days was used for R0 was calculated with a shift of 7 days. The necessary herd immunity level was counted as (1-1/R0)*100%.

## Data availability statement

The data presented in the study are deposited in the GenBank repository, accession number OP584857.

## Ethics statement

The studies involving human participants were reviewed and approved by Ethics Committee of “National Research Centre for Epidemiology and Microbiology Named After Honorary Academician N. F. Gamaleya” of the Ministry of Health of the Russian Federation, Moscow, Russia. The patients/participants provided their written informed consent to participate in this study.

## Author contributions

VG, DL, and AG conceived of the study. OB, ES, AT, and AK were responsible for the patient recruitment, collected the clinical data, and organized the patient specimen collection. OZ, IVG, TO, NK, DZ, AO, VK, EB, AB, and LV performed antigen preparation and the ELISA immunoassays, ID, AI, AS, DG, SD, and AnB performed virus neutralization assay, AP and DK carried Vaccinia virus sequencing out the NGS data analysis, VG, DO, ID, carried out the immunologic data analysis, VG and DO drafted the manuscript. NK, AO, EB, AB, and LV performed sample preparation and specimen logistics, and completed and verified the database. VG, ID, OZ, ArT, ES, AT, DV, DK, AK and DL contributed to the study design. VG, DL and AG contributed to the funding acquisition. VZ, DS, AS, SL, ER, SB, BN, DL, and AG provided valuable comments and suggestions. All authors contributed to the article and approved the submitted version.

## Funding

Ministry of Health of Russia and Moscow Healthcare Department.

## Conflict of interest

The authors declare that the research was conducted in the absence of any commercial or financial relationships that could be construed as a potential conflict of interest.

## Publisher’s note

All claims expressed in this article are solely those of the authors and do not necessarily represent those of their affiliated organizations, or those of the publisher, the editors and the reviewers. Any product that may be evaluated in this article, or claim that may be made by its manufacturer, is not guaranteed or endorsed by the publisher.
