# Supplementary material for: Estimation of anti-orthopoxvirus immunity in Moscow residents and potential risks of spreading Monkeypox virus
Source: Front Immunol. 2022 Nov 16;13:1023164. doi: 10.3389/fimmu.2022.1023164 (PMC9709467; doi:10.3389/fimmu.2022.1023164)
Supplement: Supplementary file 1 [file DataSheet_1.docx]

**Supplementary**

**Estimation of residual immunity to smallpox vaccine in Moscow residents and potential risks of spreading Monkeypox virus**

**Vladimir A. Gushchin^1,2,*^, Darya A. Ogarkova^1^, Inna V. Dolzhikova^1^, Olga V. Zubkova^1^, Igor V. Grigoriev^1^, Andrei A. Pochtovyi^1,2^, Anna A. Iliukhina^1^, Tatiana A. Ozharovskaia^1^, Nadezhda A. Kuznetsova^1^, Daria D. Kustova^1^, Artem Y. Shelkov^1^, Denis I. Zrelkin^1^, Alina S. Odintsova^1^, Daria M. Grousova^1^, Vladislav Y. Kan^1^, Sona A. Davtyan^1^, Andrei E. Siniavin^1^, Elizaveta D. Belyaeva^1^, Andrei G. Botikov^1^, Arina A. Bessonova^1^, Lyudmila A. Vasilchenko^1^, Daria V. Vasina^1^, Denis A. Kleymenov^1^, Egor A. Slutskiy^3^, Artem P. Tkachuk^1^, Olga A. Burgasova^1,3,4^, Svetlana Y. Loginova^5^, Evgeny V. Rozhdestvensky^5^,Dmitry V Shcheblyakov^1^, Alexander N. Tsibin^3^, Andrey G. Komarov^3^, Vladimir I. Zlobin^1^, Sergei V. Borisevich^5^, Boris S. Naroditsky^1^, Denis Y. Logunov^1^, Alexander L. Gintsburg^1,6^**

1 Federal State Budget Institution “National Research Centre for Epidemiology and Microbiology Named After Honorary Academician N. F. Gamaleya” of the Ministry of Health of the Russian Federation, Moscow, Russia, 2 Department of Virology, Biological Faculty, Lomonosov Moscow State University, Moscow, Russia, 3 Moscow Healthcare Department, Moscow, Russia, 4 Department of Infectious Diseases with the Courses of Epidemiology and Phthisiology, Рeoples Friendship University of Russia (RUDN) University, Moscow, Russia, 5 48-Central Research Institute of the Ministry of Defence of the Russian Federation, Moscow, Russia, 6 Department of Infectiology and Virology, Federal State Autonomous Educational Institution of Higher Education I.M. Sechenov, First Moscow State Medical University of the Ministry of Health of the Russian Federation (Sechenov University), Moscow, Russia

*Correspondence to Dr. Vladimir A. Gushchin, e-mail: wowaniada@gmail.comwowaniada@gmail.com


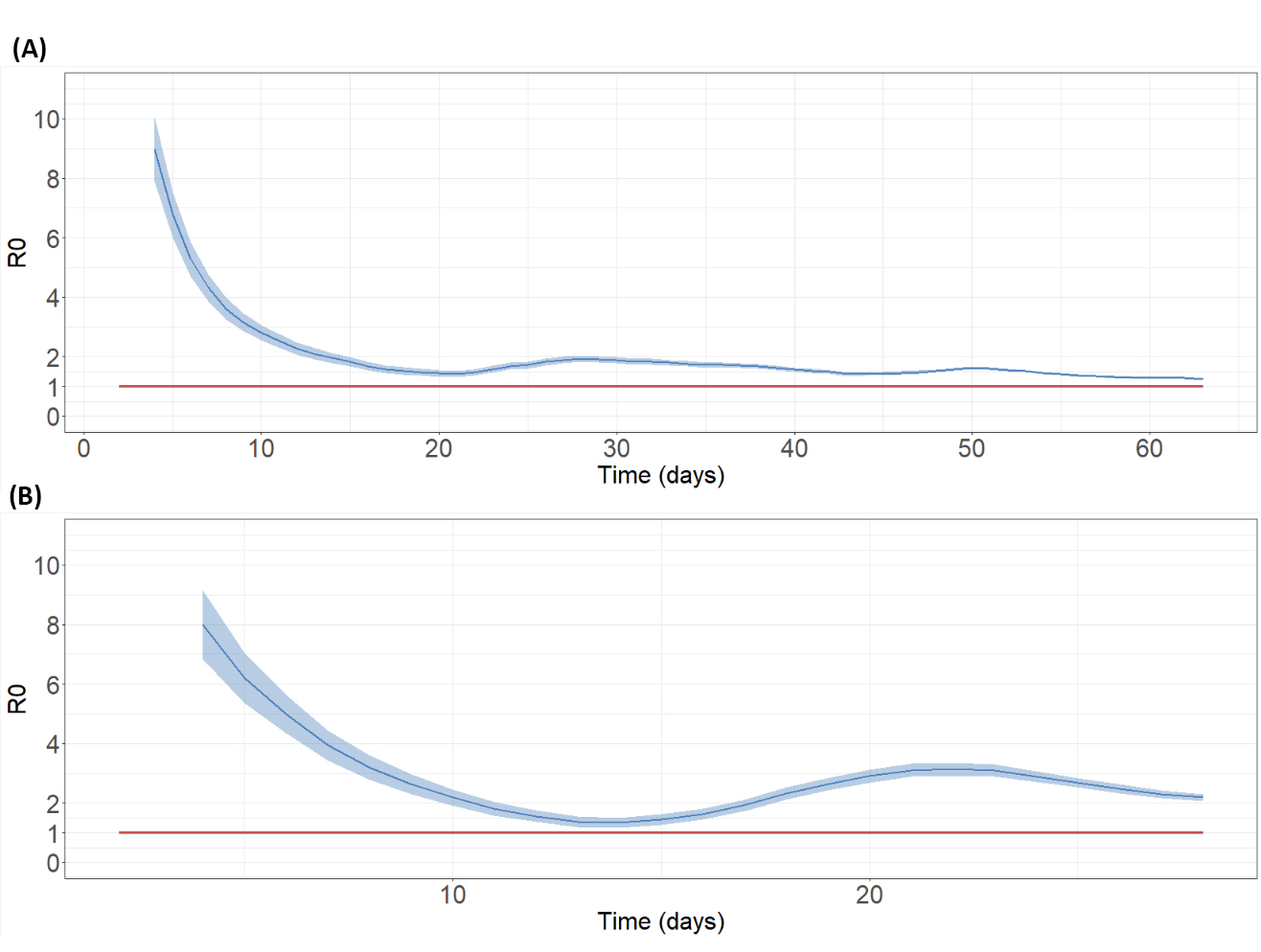


**Figure S1.** Monkeypox virus R0, counted with worldwide daily cases (А) and US cases (В). Blue line shows weekly R0 and 95% confidence level, red line shows threshold value of R0 = 1. The countdown starts from the day when the 7–day moving average number of cases exceeded 10: 05.20.22 for global statistics and 22.06 for the United States. incubation period of 9.8 days with a standard deviation of 4 days was used for calculation (16). The maximum value of R0 was 8.97 for the world and 7.99 for the USA. The median was 1.61 and 2.63 for the world and the USA, respectively, and the average was 2.01 (95% CI: 1.67 – 2.35) and 2.88 (95% CI: 2.24 – 3.51).


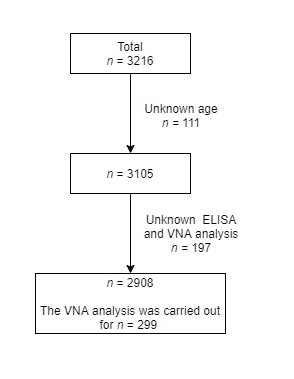


**Figure S2. Database processing**
